# A One-Bit Programmable Multi-Functional Metasurface for Microwave Beam Shaping

**DOI:** 10.3390/mi14112011

**Published:** 2023-10-29

**Authors:** Wu Zhang, Jiahan Lin, Zitao Zheng, Yusong Gao, Jifang Tao, Wenli Shang, Meng Zhang

**Affiliations:** 1 School of Physics and Material Science, Guangzhou University, Guangzhou 510006, China; zhangwu@gzhu.edu.cn (W.Z.); 2112119048@e.gzhu.edu.cn (J.L.);; 2 School of Information Science and Engineering, Shandong University, Jinan 250100, China; 3 School of Electronics and Communication Engineering, Guangzhou University, Guangzhou 510006, China; 4 Key Laboratory of On-Chip Communication and Sensor Chip of Guangdong Higher Education Institute, Guangzhou 510006, China

**Keywords:** metasurface, programmable, vortex beam generation, tunable focus

## Abstract

In this paper, we demonstrate a multi-functional metasurface for microwave beam-shaping application. The metasurface consists of an array of programmable unit cells, and each unit cell is integrated with one varactor diode. By turning the electrical bias on the diode on and off, the phase delay of the microwave reflected by the metasurface can be switched between 0 and π at a 6.2 GHz frequency, which makes the metasurface 1-bit-coded. By programming the 1-bit-coded metasurface, the generation of a single-focus beam, a double-focus beam and a focused vortex beam was experimentally demonstrated. Furthermore, the single-focus beam with tunable focal lengths of 54 mm, 103 mm and 152 mm was experimentally observed at 5.7 GHz. The proposed programmable metasurface manifests robust and flexible beam-shaping ability which allows its application to microwave imaging, information transmission and sensing applications.

## 1. Introduction

Metamaterial is an artificial material which manipulates the amplitude, phase and polarization of electromagnetic (EM) waves through arrays consisting of meta-atoms or unit cells [1]. EM waves being manipulated range from microwaves to the visible-light regime by simply designing the corresponding unit cells at a subwavelength scale. Metamaterial is thus fabricated through different methods such as PCB technology, optical lithography and E-beam lithography for microwave [2], THz wave [3] and visible-light control [4], correspondingly. Different unit cell structures such as the split-ring resonator [5], the fishnet [6] and the gammadion shape [7] are designed to induce different resonances of EM waves. As a result, various novel properties of EM waves including negative refraction [8], perfect absorption [9], strong optical activity [10] and extraordinary transmission [11] can be realized, which provides a fascinating platform and opens the door to a number of fascinating applications such as invisible cloaking [12], super-lenses [13] and polarization conversion [14]. Later, the two-dimensional metamaterial which consists of one layer of unit cells, also called the metasurface, was proposed. Metasurfaces have great advantages in the fabrication process compared to three-dimensional metamaterial, and thus they have received intensive research attention. Metasurfaces mainly control the wavefront of EM waves and realize various functions such as flat-lens focusing [15], beam shaping [16], flat optics imaging [17], etc. 

For dynamically manipulating EM waves, different control mechanisms such as electrical, mechanical, optical and thermal methods were investigated for a long period [18]. In recent years, programmable metasurfaces with digitally coded unit cells, which link the digital world and the physical world, began to attract many researchers’ attention for their application in EM wave manipulation [19,20,21]. In a programmable metasurface, each unit cell is coded to induce a digitized phase response for an EM wave, for example, 0 or π, and the unit cell is considered as being coded as “0” or “1”, resulting in a 1-bit programmable metasurface. Generally, the programmable metasurface can be coded with more than one digit. For example, a metasurface with digitized phase responses of 0, π/2, π and 3π/2 can be coded as 00, 01, 10 and 11, correspondingly. Complex coding of the unit cell was later proposed for more flexible manipulation of EM waves [22]. 

Programmable metasurfaces are usually implemented by integrating active elements in the unit cells. Mostly, a PIN diode or a varactor diode under electrical bias is used for the coding [19]. The electrical resistance or capacitance of diodes is tuned under electrical bias so that the unit cells can realize a tunable phase response to EM waves. Metasurface coding based on some other approaches has also been proposed. For example, a MEMS technology-based programmable metasurface for logic operations at terahertz frequencies was reported in [23]. Metasurfaces can also be programmed optically by integrating photodiodes into metasurface unit cells controlled by optical intensity [24]. Some special materials such as VO_2_ [25], liquid crystal [26] and graphene were recently explored for their application in programmable metasurface coding [27] as well.

Through programmable metasurfaces, different types of EM wave control have been achieved. For example, the polarization of EM wave beams can be effectively tailored through coded metasurfaces [28] and polarization-sensitive wave control was also reported [29]. The simultaneous manipulation of EM waves at different frequency bands was demonstrated through metasurface coding [30]. EM wave shaping through programmable metasurfaces has been demonstrated in different aspects such as beam focusing [31], multi-beam generation [32], airy beam manipulation [33], vortex beam generation [34], etc. Holographic imaging through a programmable metasurface was also reported [35]. Furthermore, metasurfaces have a broad application in the wireless communication field and have a great potential to reduce the path loss in microwave signal propagation in a cost-effective and energy-efficient manner [36]. In this paper, we propose a 1-bit programmable multi-functional metasurface for microwave beam shaping. A varactor diode is used in a metasurface unit cells to manipulate the phase-reflected microwave between 0 and π. Through phase programming, we experimentally demonstrate multiple functions of the proposed metasurface. First, a focused microwave beam with tunable focal length is realized; second, a microwave beam with double focal spots is obtained; finally, a focused vortex beam is generated through the proposed metasurface.

## 2. Materials and Methods

**Figure 1:**
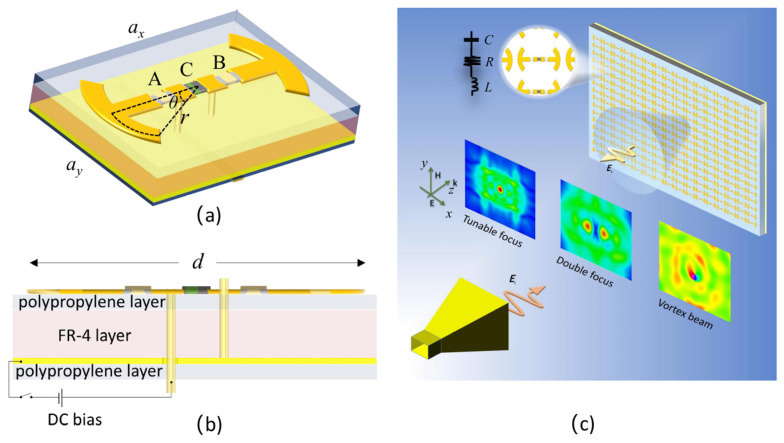
(**a**) Perspective view and (**b**) side view of the designed programmable metasurface unit cell; (**c**) the reflected wave from the metasurface is programmed to different beam shapes.

The proposed unit cell of the programmable metasurface is illustrated in Figure 1. The unit cell substrate was chosen to be a flame retardant (FR)-4 printed circuit board (PCB) layer, which was coated with a polypropylene layer on the top, a middle copper layer and another polypropylene layer on the bottom. On the substrate, two symmetric arc copper strips were connected to each other through two capacitors A and B and a varactor diode C in between. The varactor diode was biased with a direct-current (DC) voltage through a bias strip line at the backside of the substrate. The lattice constants *a*_x_ and *a*_y_ of the unit cell were 20 mm and 15 mm, respectively, and the thicknesses of the FR-4 layer and polypropylene layer were 1.1 mm and 0.21 mm, respectively. The two arc strips were separated by a distance of *d* = 16 mm and had a strip width of 2 mm. The arc had a radius *r* of 3 mm with a half central angle *θ* = 30°. A and B had a capacitance of 0.4 pF and a length of 1 mm, while C was modeled as the varactor diode SMV2020-079L, having a length of 1.27 mm with a tunable capacitance. By tuning the capacitance of the varactor diode, we demonstrate here a 1-bit coded metasurface which realizes differently shaped EM wave beams such as a beam with tunable focus, a beam with double focus and a focused vortex beam as illustrated in Figure 1c. 

**Figure 2:**
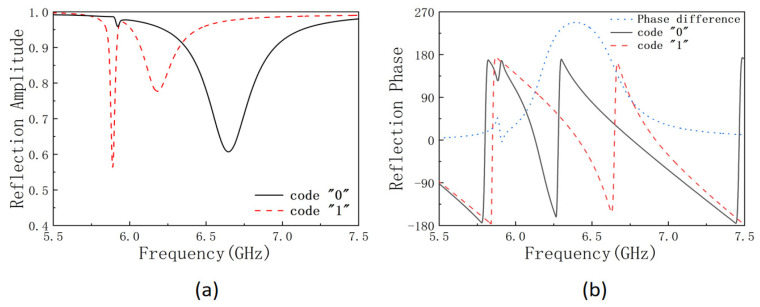
(**a**) The x-polarized reflected electrical field intensity and (**b**) the reflected phase of the metasurface when the unit cell is uniformly coded at “0” (black solid line) or “1” state (red dash line), and the phase difference between the two states (blue dot line).

We first numerically simulate the *S*-parameter of the metasurface using CST microwave studio. The periodic boundary condition is applied for the unit cell in both the x and the y directions. A linearly polarized plane wave is incident on the unit cell with polarization in the x direction. The permittivities of the FR-4 and polypropylene materials are set at 4.48 and 4.8, respectively. The copper has a conductance of 5.8 × 10^7^ S/m. The varactor diode is modeled as an LCR series circuit as illustrated in the insertion of Figure 1c, in which the effective inductance *L*_eff_ = 0.7 pH and effective resistance *R*_eff_ = 2.5 Ω. By switching the biased voltage between 0 V and 20 V, the effective capacitance of the varactor diode is switched between 3.2 pF and 0.35 pF, which codes the unit cell as “0” and “1”, correspondingly. The reflected microwave amplitudes between 5.5 GHz and 7.5 GHz from the metasurface with unit cells uniformly coded as “0” and “1” are plotted in Figure 2a with a black solid line and a red dash line, respectively. In Figure 2b, the reflected phase difference between the two coded metasurfaces are plotted with a blue dot line, and a phase difference of 180° is obtained at a frequency *f* = 6.2 GHz and *f* = 6.6 GHz. At *f* = 6.6 GHz, the reflected amplitude of the “0” coded metasurface is only 62%, which is relatively low and much smaller than the amplitude of 97% for the “1” code metasurface, while at *f* = 6.2 GHz, the reflection amplitude of the “0” code metasurface is 80%, which is relatively high and is comparable with the amplitude 95% for the “1” code metasurface. Therefore, we program the metasurface at *f* = 6.2 GHz or wavelength at *λ* = 48 mm with proper code distribution to achieve different beam shaping applications. Here, we demonstrate the beam shaping application by generating the beam with tunable focus, beam with double focus and focused vortex beam successively. 

According to the beam focusing principle, which is the same as that for designing a zone plate, the wave reflected from unit cells at different locations should be in phase at the focal spot when the plane wave is incident on the metasurface. Therefore, the compensated phase of the unit cell at the coordinate (*x*, *y*) can be expressed as

(1) $$\Delta \varphi (x,y)=\frac{2\pi }{\lambda }\left(\sqrt{{\left(F_{x}-x\right)}^{2}+{\left(F_{y}-y\right)}^{2}+F_{z}^{2}}-F_{z}\right)+2n\pi ,$$

where λ is the incident wavelength, $F_{x}$ and $F_{y}$ represent the focus position in the x and the y direction on the focal plane, $F_{z}$ represents the focal length and *n* is an integer.

For obtaining a beam with multiple foci, the required electric field distribution of the metasurface is set as a superposition of multiple single focused electric field vectors, and the corresponding compensated phase distribution can be expressed as

(2) $$\Delta \varphi (x,y)=arg\left\{\sum_{n=1}^{N}\left[A_{n}exp\left(-j\frac{2\pi }{\lambda }\left(\sqrt{{\left(F_{x}-x\right)}^{2}+{\left(F_{y}-y\right)}^{2}+F_{z}^{2}}-F_{z}\right)\right)\right]\right\}+2n\pi ,$$

where *N* is the number of foci, $A_{n}$ stands for the electric field intensity weight at the *n*th focal spot.

For the vortex beam, the topological charge number is defined as the number of phase twists at one wavelength propagation. This induces an orbital angular momentum and has a rotational phase profile associated with azimuth angle, which can be expressed as

(3) $$\theta (x,\;y)=arctan\left(\frac{y}{x}\right).$$

The compensated phase for the focused vortex beam is obtained by combing the phases of the vortex beam and the focus beam, which is expressed as

(4) $$\Delta \varphi (x,y)=\frac{2\pi }{\lambda }\left(\sqrt{{\left(F_{x}-x\right)}^{2}+{\left(F_{y}-y\right)}^{2}+F_{z}^{2}}-F_{z}\right)+l\theta (x,\;y)+2n\pi ,$$

where *l* is the topological number. 

It should be noted that the phases acquired from above equations are continuous and need to be digitized for unit cell coding, which can be expressed as

(5) $$\Delta \varphi_{b}(x,y)=\left\{\begin{array}{c} 0,\;\;\;\;0\leq \Delta \varphi \left(x,y\right)<\pi \\ \pi ,\;\;\;\;\;\;\;\;\;\;\;\;\;\;\;\;\;\mathrm{otherwise} \end{array}\right.$$

for a 1-bit coded metasurface, where $\Delta \varphi_{b}$ is the digitized phase. The metasurface unit cell is coded as “0” when $\Delta \varphi_{b}$ = 0 and coded as “1” when $\Delta \varphi_{b}$ = π. 

## 3. Simulation Results and Discussions

**Figure 3:**
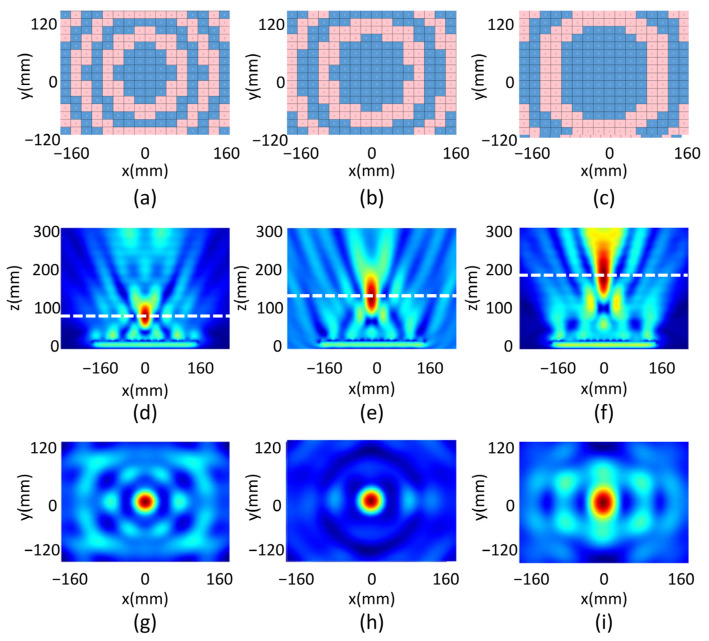
The code distribution for the beam focusing function of designed focal length at (**a**) 50 mm, (**b**) 100 mm and (**c**) 150 mm; the calculated electrical field intensity distribution of the reflected microwave in the xz plane for the designed focal length at (**d**) 50 mm, (**e**) 100 mm and (**f**) 150 mm; the reflected electrical field in the xy plane for the designed focal length at (**g**) 50 mm, (**h**) 100 mm and (**i**) 150 mm.

To realize the beam shaping functions mentioned above, we design the metasurface consisting of 16 × 16-unit cells, with a total size of 320 × 240 mm. A focus beam with tunable focal length is first demonstrated by setting digitized phase distribution of the unit cells according to Equation (1), in which *F*_x_ and *F*_y_ are set at zero so that the focal spot is above the center of the metasurface. *F*_z_ is set at 50 mm, 100 mm and 150 mm, respectively, to realize three different focal lengths. The code distribution of the unit cells is as shown in Figure 3a–c for the three different focal lengths, where the blue color stands for code “0” and the red color stands for code “1”. The corresponding electrical field intensity of the reflected microwave by the metasurface at 6.2 GHz is plotted in Figure 3d–f for the three focal length designs. Obvious foci with focal lengths of 64 mm, 116 mm and 176 are observed by tracing the distance between the metasurface and the point in the focal spots with the maximum intensity. The measured focal lengths deviate from the designed values, which is resulted from the error when phase is digitized in Equation (5) compared to the continuous phase calculated from Equation (1), as well as from the reflected intensity inequality of code “0” and code “1” unit cells. 

The focal planes for the three focal length cases are plotted in Figure 3g–i, respectively. The full width at half maximum (FWHM) value in the x and the y directions, FWHM_x_ and FWHM_y_, for the three foci are summarized in Table 1. For the metasurface with a designed *F*_z_ = 50 mm case, the corresponding FWMH value of the focal spot ((FWHM_x_ = 39.5 mm and FWHM_x_ = 41 mm) is less than the incident wavelength (*λ* = 48 mm for *f* = 6.2 GHz), indicating a sub-wavelength focus. The FWMH is smaller for a shorter focal length because the numerical aperture is lager, focusing the wave into a smaller spot. A difference between FWHM_x_ and FWHM_y_ is observed for all three cases, which is due to the anisotropic design of the unit cell. Finally, we evaluate the focal efficiency as

(6) $$\eta_{s}=\frac{{\left|E_{FWMH\times 3}\right|}^{2}}{{\left|E_{total}\right|}^{2}}\times 100\%,$$

where $E_{FWMH\times 3}$ is the energy in the area of triple-sized FWHM around the focus in the focal plane, and $E_{total}$ is the energy of the wave incident on the entire metasurface. As listed in Table 1, the focal efficiency is 14.6%, 21.1% and 36.3% for the focal length *F*_z_ = 50 mm, 100 mm and 150 mm cases, correspondingly. The efficiency increases with the focal length, because at a shorter focal length, a larger angle is required for the incident wave to be bent to the focal spot, which results in a higher energy loss. 

**Figure 4:**
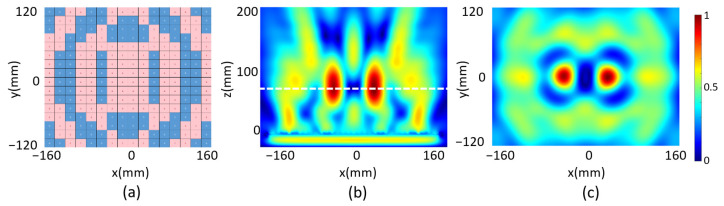
(**a**) Code distribution of the programmable metasurface with double focus; (**b**) reflected electrical field intensity in the xz plane and (**c**) reflected electrical field intensity in the focal plane.

The beam with double focus is realized through the programmable metasurface by setting *N* = 2 in Equation (2). Assuming *F*_x1_ = −40 mm, *F*_y1_ = 0 and *F*_z1_ = 100 mm for Focus 1, and *F*_x2_ = 40 mm, *F*_y2_ = 0 and *F*_z2_ = 100 mm for Focus 2, and setting the electric field intensity weight $A_{n}$ = 1 for both foci, we obtain the required phase distribution on the metasurface from Equation (2), which is then digitized according to Equation (5) and as shown in Figure 4a. The reflected electrical field intensity in the xz plane at *y* = 0 is plotted in Figure 4b, and two focal spots are observed. The corresponding focal lengths in the z directions *F*_z1_ and *F*_z2_ are both 74 mm when measuring the distance of the two focal spots with maximum electrical field to the metasurface. The measured focal lengths deviate from the designed values, which should be also due to the phase error caused by the digitization and the inequality of the reflected intensity between the “0”- and “1”-coded unit cells mentioned earlier. The focal plane is plotted in Figure 4c, which manifests two identical focal spots with FHWM_x_ = 42 and FHWM_y_ = 46 mm. The sizes of the two focal spots are comparable with the incident wavelength. It is worth pointing out that the focal spots with different intensity can also be realized by simply setting different electric field intensity weights for different spots, which is not discussed here. 

**Figure 5:**
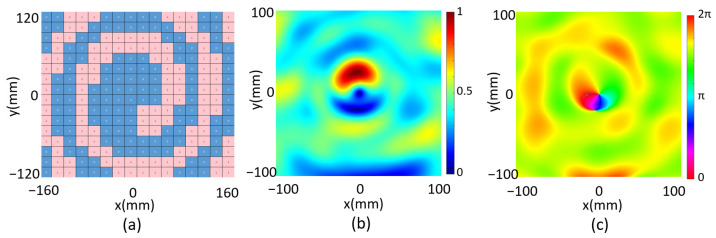
(**a**) Metasurface code distribution, (**b**) the calculated reflected electrical field intensity and (**c**) the reflected electrical phase of the focal vortex beam.

We finally investigate a focused one-order vortex beam through the programmable metasurface, which requires a reflected phase distribution on the metasurface according to the Equation (4). By setting *F*_x_ = *F*_y_ = 0, *F*_z_ = 70 mm and *l* = 1, we obtain the unit cell phase distribution which is then digitized as shown in Figure 5a. The reflected electrical field intensity and phase in xy plane are both calculated at z = 70 mm as shown in Figure 5b,c, correspondingly. The electrical field intensity is zero at the center, and the reflected phase distribution manifests a spiral behavior, indicating a vortex beam is generated. As a conclusion, the multi-functional metasurface is numerically demonstrated by programming the metasurface for beam shaping of the microwave into a single-focus beam, a double-focus beam and a focused vortex beam. 

## 4. Experimental Results and Discussions

**Figure 6:**
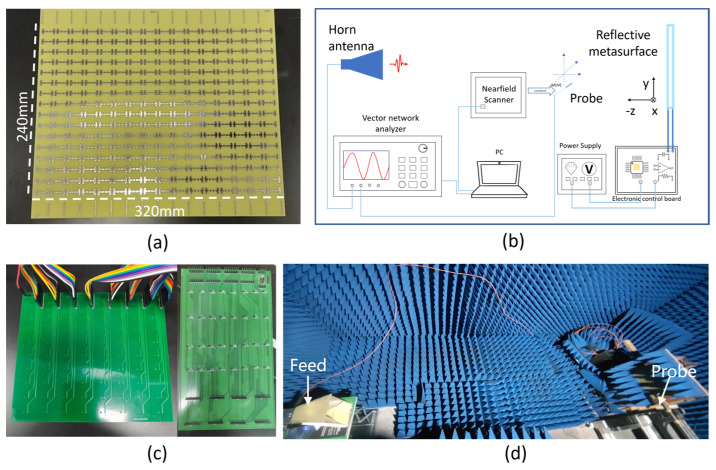
(**a**) Fabricated programmable metasurface; (**b**) metasurface control circuit; (**c**) schematic of experimental setup and (**d**) experiment environment.

The programmable metasurface was fabricated for experimental investigation of the multi-functional beam shaping on the microwave, which consists of 16 × 16-unit cells as shown in Figure 6a. The metasurface substrate is made of 1.1 mm thick FR-4 layer coated with a thin polypropylene layer on the top and a copper layer at the bottom. The unit cell structure is the same as describe above, in which a varactor diode (SMV2020-079L) is integrated. Two pins of the varactor diode are connected to the strip lines at the backside of the metasurface. The strip lines of all unit cells are connected to a control circuit consisting of four decoders (CD74HC238EG4) and one microcontroller (STM32) as shown in Figure 6b, which is used to electrically bias each varactor diode independently. The switching time for reprogramming the metasurface mainly depends on the control circuit and is estimated within 1 μs. The bias is either 0 V or 20 V to code the corresponding unit cell as “0” or “1” condition. The experimental setup for scanning the reflected wave intensity is illustrated in Figure 6c, which was in an anechoic chamber to eliminate the microwave reflected from the environment. One port of the vector network analyzer (Keysight, N5225A) was connected a horn antenna to emit a microwave with linear polarization. The polarization direction can be changed by simply rotating the horn antenna. The metasurface sample holder was placed about 2.5 m away from the horn antenna and the microwave was normally incident on the metasurface. An electrical field probe connected to an electrical field scanner (Linbou Nearfield Technology, Shenzhen, China) was used to scan the electrical field. The electrical probe behaves as a dipole antenna, and the instant electrical current induced by the time-varying electrical field of the EM wave was recorded by the electrical field scanner, which is synchronized with the signal emitted from the vector network analyzer. Therefore, both the intensity and phase in front of the metasurface can be obtained. The step size of the scanning was set at 6 mm, which is eight times smaller than the targeted wavelength and is therefore fine enough for characterizing the electrical field distribution. 

**Figure 7:**
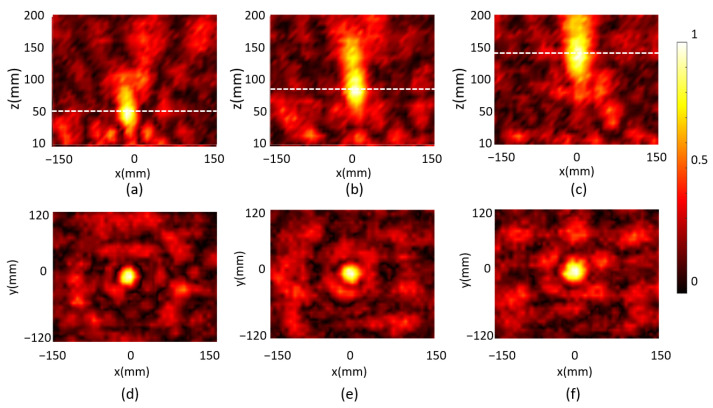
The measured reflected electrical field intensity in the xz plane and in the xy plane for the designed focal length of (**a**,**d**) 50 mm; (**b**,**e**) 100 mm and (**c**,**f**) 150 mm, correspondingly.

The incident wave was first scanned without the metasurface on the sample holder, which is noted as *E*_incident_, and contained both electrical field intensity and phase information. The electrical field *E*_total_ was then measured when the metasurface was on the sample holder. The reflected electrical field was then obtained as *E*_reflected_ = *E*_total_ − *E*_incident_. We first programmed the metasurface according to the code distribution as shown in Figure 3a–c to demonstrate a focus beam with a tunable focal length. The reflected electrical field in front of the metasurface was scanned in the frequency range between 4 GHz and 8 GHz. To clearly describe the scanned area, we defined the measuring plane parallel with the metasurface as the xy plane, and assumed the metasurface was located at z = 0 with the center at x = 0 and y = 0. We first scanned the electric field intensity of the metasurface in the xz plane at y = 0. The scanned area was from −150 mm to 150 mm in the x direction and from 10 mm to 200 mm in the z direction. At the three programmed states, three different focal patterns were well observed at 5.7 GHz as shown in Figure 7a–c. The corresponding focal lengths *F*_z, measure_ were measured at 54 mm, 103 mm and 152 mm, respectively, which demonstrated that the focal length of the metasurface can be significantly tuned. The corresponding focal planes at the three programmed states were then scanned as shown in Figure 7d–f, respectively, and the scanned area was 300 × 240 mm. The FWMH in both the x and the y directions and the focal efficiency $\eta_{s}$ were analyzed and summarized in Table 2. The measured focal spot was in a sub-wavelength size with focal efficiency above 20% for all tuned focal length cases, which demonstrated a good focal performance. It was also noticed that the intensity patterns in the focal planes of the three cases were not symmetric. This was mainly because the electrical field probe was suspended by a plastic rod in front of the metasurface, which disturbed both incident and reflected wave. The measured focal lengths were deviated from simulation results due to several reasons. First, the incident wave from the horn antenna in the experiment is not an ideal plane wave, which may induce phase mismatch with the simulation results. Second, in the simulation, each unit cell only contains one strip line for the electrical bias purpose, while in the fabricated metasurface, the unit cell may contain more than one strip line which are extended from neighboring unit cells. This affects the unit cell resonant frequency and phase response. Third, the contact resistance between the soldered varactor diode and the bias strip lines was not considered in the simulation, which also affects the unit cell resonant frequency and phase response. In addition, the probe for measuring the field inevitably disturbs the incident and reflected field. 

**Figure 8:**
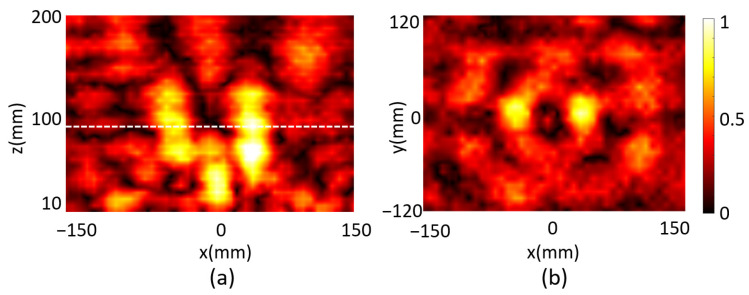
The measured reflected field intensity in (**a**) the xz plane and (**b**) the focal plane of the metasurface designed for the double-focus beam.

**Figure 9:**
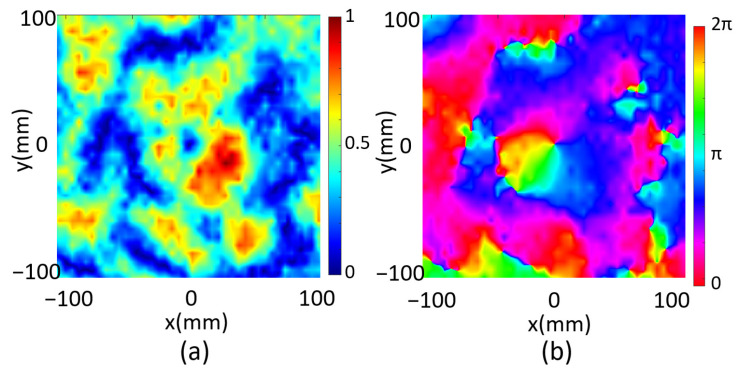
(**a**) The measured reflected electrical field intensity and (**b**) the phase distribution of the metasurface designed for a 1-order vortex beam.

Next, we scanned the reflected electrical field of the metasurface programmed for double-focus beam shaping. Two focal spots were observed on the xz plane for the microwave at *f* = 5.7 GHz as shown in Figure 8a, which is about 80 mm in front of the metasurface. The electrical field on the xy plane with z = 80 mm was then scanned and the reflected electrical field intensity was plotted in Figure 8b. It was measured that FWHM_x1_ = 39 mm and FWHM_y1_ = 50 mm for one spot and FWHM_x2_ = 39 mm and FWHM_y2_ = 56 mm for the other, which demonstrated the two spots are almost identical. We finally programmed the metasurface with code distribution as shown in Figure 5a for focal vortex beam generation. The reflected field intensity and the phase of the microwave were scanned and plotted in Figure 9a,b, respectively, at 68 mm above the metasurface, which manifests an obvious one-order vortex beam pattern. A point with zero intensity was measured at the center of the area, and the spiral phase distribution further confirms the vortex beam generation. 

## 5. Conclusions

In conclusion, we demonstrated an multi-functional programmable metasurface to realize different microwave beam shaping applications. The metasurface is 1-bitcoded by controlling the capacitance of the varactor diode in the metasurface unit cells. Through proper code distribution programming, the beam focusing at three different focal lengths of 54 mm, 103 mm and 152 mm was experimentally demonstrated at the microwave of 5.7 GHz, with all focal spots at a sub-wavelength scale. A beam with double focus was also realized with the spot size comparable with the incident wavelength. Finally, a one-order focused vortex beam was generated and a reflected field with spiral phase distribution and zero intensity at the center was observed experimentally. The proposed programmable metasurface manifests robust and flexible multifunctional microwave beam control, which allows its application for microwave imaging, information transmission and sensing applications. 

## Figures and Tables

**Table 1:** The numerically calculated focal spot parameter for metasurface programmed at different designed focal lengths.

Designed *F*_z_ (mm)	Simulated *F*_z_ (mm)	Simulated FWMH_X_ (mm)	Simulated FWMH_Y_ (mm)	$\mathrm{Simulated}\;\eta_{s}$ (%)
50	64	39.5 (0.82 *λ*)	41 (0.85 *λ*)	14.6
100	116	44 (0.92 *λ*)	47 (0.98 *λ*)	21.1
150	176	50 (1.04 *λ*)	74.5 (1.55 *λ*)	36.3

**Table 2:** The experimental results of the reflected focus beam with different designed focal length.

Designed *F*_z, design_	Measured *F*_z, measure_	Measured FWMH_X_	Measured FWMH_y_	$\mathrm{Measured}\;\eta_{s}$
50 mm	54 mm	30 mm (0.57 *λ*)	31 mm (0.59 *λ*)	24.5
100 mm	103 mm	40 mm (0.76 *λ*)	34 mm (0.64 *λ*)	20.3
150 mm	152 mm	46 mm (0.87 *λ*)	41 mm (0.78 *λ*)	25.0

## Data Availability

Raw data presented in this study are available on request from the corresponding author.
